# The Hull Infirmary

**Published:** 1887-02-12

**Authors:** Henry Simpson

**Affiliations:** Chairman of the Board of Management. The Hull Royal Infirmary, Hull


### The Editor's Letter-Box.

[Our Correspondents are reminded that prolixity is a great bar to publication, and that brevity of style and conciseness of statement greatly facilitate early publication.]

### THE HULL INFIRMARY; THE COST OF HOSPITAL BEDS: AN EXPLANATION.

In *The Newcastle Chronicle* of the 15th inst., a report from the authorities of the local Infirmary was published. In this report was included a comparative statement, showing the respective cost of beds in various specified hospitals. This statement has been copied, and will probably receive a wide circulation, and on this account I wish to draw attention to the position which the Hull Royal Infirmary holds in the list.

The figures given are correct, but their value is greatly affected by the existing condition of things in 1885, the time they were taken. The Infirmary is being extended and rearranged, and the whole building was, during the period referred to, more or less in the hands of workmen, which gave results which certainly ought not to have been used for purposes of comparison. The statement showed Hull to have ninety-six beds occupied in 1885, while the three years preceding gave an average of 118, and this year, though still in the midst of works, we have risen to 125. As your paper circulates chiefly amongst those interested in hospitals, I venture to ask you to give early admission to this explanation.

I beg also to send copy of a pamphlet, which will show what we are doing here in the way of hospital extension and improvement.

Henry Simpson,
Chairman of the Board of Management.

The Hull Royal Infirmary, Hull.
January 22, 1887.

